# A novel *exo*-lytic and disaccharide-yielding glycosaminoglycan lyase from a marine-derived polysaccharide-degrading actinobacterium *Microbacterium* sp. strain WS15

**DOI:** 10.3389/fmicb.2025.1680841

**Published:** 2025-11-17

**Authors:** Peng Li, Zaichao Ma, Ruyi Zou, Jingyan Gu, Ningning Shan, Wenjun Han, Hailong Wang, Fuchuan Li, Haining Tan

**Affiliations:** 1 National Glycoengineering Research Center, Shandong Key Laboratory of Carbohydrate Chemistry and Glycobiology, NMPA Key Laboratory for Quality Research and Evaluation of Carbohydrate-based Medicine, State Key Laboratory of Microbial Technology, Shandong University, Qingdao, China; 2 United Post-Graduate Education Base of Shandong University, Jinan Enlighten Biotechnology Co. Ltd., Jinan, China; 3 Department of Hematology, The Second Hospital of Shandong University, Jinan, China

**Keywords:** active site residue, action mode, GAG lyase, molecular docking, sulfation

## Abstract

**Introduction:**

Glycosaminoglycan (GAG) lyases play key roles in preparing oligosaccharides, treating human diseases, and learning relationships between complex structures and various functions of carbohydrates. An *endo*-lytic enzyme will depolymerize GAGs randomly and finally produce a series of size-defined oligosaccharide fractions, whereas an *exo*-lytic lyase can usually yield the sole final oligosaccharide products. However, it is difficult to distinguish them directly depending on protein sequences, which limits further resource exploration.

**Results and Dicussion:**

In this study, we initially isolated and identified a marine-derived polysaccharide-degrading actinobacterium, *Microbacterium* sp. strain WS15, and subsequently explored by genome sequencing and data mining, as a new candidate GAGs lyase within the eighth polysaccharide lyase (PL8) family, sharing high sequence identity with characterized *endo*-type GAG lyases. The recombinant proteins of TT16 were optimal at 50 °C and pH 7.0, respectively, and active against multiple polysaccharide substrates, including both unsulfated GAG (e.g., hyaluronate) and sulfated GAGs (e.g., chondroitin sulfate A–E types), implying a broad spectrum based on sulfation tolerance. However, TT16 was novel for predominantly yielding unsaturated disaccharides as an *exo*-type lyase, rather than an *endo*-type lyase, with the smallest substrate being associated with tetrasaccharides, suggesting the potential in disaccharide preparation. Comparative enzymatic analyses indicated that all the biochemical characteristics and catalytic properties were determined by the TT16 protein rather than the additional protein tag. Moreover, protein structure modeling followed by molecular docking revealed that protein TT16 showed low binding energies with various donors, and its catalytic cavity was large and flexible to accommodate either two unsulfated hyaluronate disaccharides or a chondroitin sulfate E (CSE) tetrasaccharide with four sulfate groups as a donor, implying a structural basis suitable for the disaccharide-yielding type. Furthermore, the motif of Tyr284, Asn225, and His275 also provided the catalytic basis for *β*-elimination, while residues Ala71 and Arg219 might be essential for the novel *exo*-lytic mode.

**Significance:**

This study provided the protein TT16 as a novel *exo*-lytic tool for preparing unsaturated GAG disaccharides and the potential *exo*-lytic mechanism, which will benefit the initial enzyme identification and further property improvements

## 1 Introduction

Glycosaminoglycans (GAGs) are acidic and linear polysaccharides that are composed of repeating disaccharide units and linked by glycosides. The polymers exist widely in the connective tissue of animals, and most of them are covalently bonded with core proteins to form proteoglycans *in vivo* (Norbert and Merton, 2001; Xing et al., 2020). GAGs are diverse in structures, and proteoglycans play various biological functions in many physiological or pathological processes, for example, adhesion, anticoagulation, development, protection, and recognition of cells (Norbert and Merton, 2001; Renato and Liliana, 2015). Numerous studies have discovered that the length of sugar chains, the arrangement of monosaccharide residues, and the degree and location of sulfation in sugar units have co-determined the capabilities of GAG chains, for example, the binding strength to various protein factors, and thus affect the biological activities of proteoglycans in which GAGs reside (Timothy and Amanda, 1992; Mary et al., 1999; Liliana and Roland, 2010).

According to the sulfation property of disaccharide units, GAGs can be roughly divided into polysaccharides without sulfate groups, for example, hyaluronic acid (HA) and chondroitin; and polysaccharides with sulfate groups, for example, chondroitin sulfate (CS), heparin (HP), heparin sulfate (HS), dermatan sulfate (DS), keratan sulfate (KS), and other categories (Michiru et al., 2006). The polysaccharide HA is simple in structure for being unsulfated, composed of repeating disaccharide units of D-glucuronic acid (GlcA) and N-acetylglucosamine (GlcNAc), and linked by internal β-1,3-glycosidic bonds and external β-1,4-glycosidic bonds (Bo et al., 2012). Chondroitin, another unsulfated polysaccharide, can be sulfated to form the polymer CS, the backbone of which is composed of repeating disaccharide units of GlcA and N-acetylgalactosamine (GalNAc) rings (Michiru et al., 2006; Gallagher and Walker, 1985). Moreover, due to the sulfation degrees and the sulfation sites, the CS polysaccharide can be further divided into various types (Kazuyuki et al., 2003), for example, chondroitin sulfate A (CS-A), C (CS-C), and E (CS-E) are individually sulfated on the hydroxyl groups at the C-4 and C-6 positions, and the C-4 and C-6 sites of GalNAc rings. Notably, the chondroitin sulfate D (CS-D) type is sulfated at the C-4 site of the GalNAc ring, whereas at the C − 2 site of the GlcA ring differently (Zhang et al., 2014).

Generally, the GAG polysaccharide is large in molecular weight and difficult to penetrate the cell membranes to exert its activity. However, the GAG oligosaccharide has a much smaller degree of polymerization, much lower viscosity, and is easier to absorb water, also exhibiting better solubility and a much higher biological activity. In clinical practice, oligo-CS chains could be more easily absorbed by the body through the cell membrane and exhibited a better efficacy in the treatment of rheumatoid arthritis, Alzheimer’s disease (Zhang et al., 2015; Zeng et al., 2012), atherosclerosis, and wound healing. Therefore, preparing GAG oligomers with low molecular weight (LMW) to improve bioavailability is of great scientific significance and important application value, making it one of the urgent research hotspots (Guo et al., 2014; Han et al., 2015; Elmabrouk et al., 2011; Zou et al., 2025). Accordingly, a tool-like GAG lyase should possess unique biochemical characteristics such as thermal stability, cold-adaptation, or pH tolerance, and may be useful either in the clinical field for degrading GAGs efficiently or in the food and medical fields for preparing oligosaccharides, for example, disaccharides, by depolymerizing the substrate completely to its origin (Zou et al., 2025; François et al., 2022).

GAG depolymerases are enzymes that can catalyze the depolymerization of GAG polysaccharides to produce oligosaccharides, for example, animal hydrolases will hydrolyze the linking glycoside linkages directly, while microbial lyases can eliminate both the sugar rings and depolymerize the glycoside linkages, yielding saturated and unsaturated oligosaccharide products respectively, which can be observed by a refractive index detector or by an ultraviolet detector at 232 nm accordingly. Based on the substrate spectra, GAGs depolymerases can be defined as hyaluronidase (HAase), chondroitin sulfate lyase (CSase), Heparinase, etc. (Zou et al., 2025; Wayne and Sheryl, 2000; Tripathi et al., 2012). Most HAases can degrade the HA polysaccharide to produce oligosaccharides, whereas they are also capable of digesting various CS polysaccharides to certain extents, in spite of lower degradation rates (Zou et al., 2025; Wayne and Sheryl, 2000). Furthermore, CS depolymerases can be divided into CSase ABC, CSase AC, CSase B, CSase C, and other types according to their corresponding optimal substrates (Rawat et al., 2022a; Shain et al., 1996). The GAG depolymerases have been promised with important applications in many fields, such as basic research (Zeng et al., 2012; Wang et al., 2020), clinical fields (Rani et al., 2018), and the preparation of CS oligosaccharides.

During the last decades, a variety of bacterial GAG lyases have been identified from many microorganisms, such as *Flavobacterium* sp. (Gu et al., 1995; Rye and Withers, 2002), *Bacillus* sp. (Guo et al., 2014; Kurata et al., 2015; Wang et al., 2022), *Bacteroides* sp. (Ndeh et al., 2018; Alvarez et al., 2025; Rawat et al., 2022b), *Streptomyces* sp. (Elmabrouk et al., 2011; Zou et al., 2025; Soh et al., 2001), and *Vibrio* sp. (Han et al., 2015; Wang et al., 2022; Zheng et al., 2024). In the CAZy database,^1^ characterized enzymes within the 8th polysaccharide lyase (PL8) family, for example, the HCLase M of *Microbacterium* sp. strain H14 (Sun et al., 2019) and the HCLase V of *Vibrio* sp. strain H240 (Wang et al., 2022), which generally contain one α-domain of α-helices and another β-domain of β-sheets (Hajam et al., 2023). *Endo*-type GAG lyases of the PL8 family can efficiently and broadly degrade multiple GAGs, such as HA and CS-A polysaccharides, via the β-eliminating mechanism. This process yields a series of size-defined unsaturated oligosaccharide product fractions with the degrees of polymerization of 2–8 (Wang et al., 2022; Sun et al., 2019). More than genome-sequencing and data mining, several PL8 enzymes have been further studied by gene mutation to discover mechanisms associated with their catalytic properties, for example, the important electron transfer effect of Asn and His residues for the β-elimination (Elmabrouk et al., 2011; Zheng et al., 2024; Sun et al., 2019). Recently, the research of HylA and HylB, two hyaluronidases from human commensal *Cutibacterium acnes* (Hajam et al., 2023), has reflected that amino acid substitutions can affect the degradation pattern types, in spite of sharing high sequence similarity with each other, which is consistent with the difficulty in distinguishing an *endo*-type from an *exo*-type GAG lyase by analyzing the sequence similarity directly. Therefore, it is essential to explore more GAGs depolymerase templates within the family PL8, either to discover the mechanism associated with the action mode or to explore novel *exo*-type lyase tools for preparing various oligosaccharides, which are abundant enzyme resources in microbiology (Zou et al., 2025; Lu et al., 2025).

In this research, we initially isolated a marine-derived bacterial strain, WS15, tested its polysaccharide-degrading capabilities, and finally performed the species identification by the 16S ribosomal RNA (rRNA) gene sequence. Next, through genome sequencing, further data mining, and heterologous gene expression, we purified the recombinant C-terminal fragment (rCTF)-TT16 (or rTT16) and enzymatically characterized the biochemical properties. We also analyzed the action mode of the recombinant enzymes against various GAG substrates and performed structure identification corresponding to oligosaccharide products through gel filtration, high-performance liquid chromatography (HPLC), mass analyses, and ^1^H-nuclear magnetic resonance (NMR). Finally, we homology-modeled the protein structure of TT16 and then molecule-docked it with substrates of both unsulfated and sulfated GAG tetrasaccharides. This was done to discover the mechanism associated with the catalytic properties, focusing on the broad spectrum, the novel disaccharide-yielding property, and the protein-structure basis for the unique *exo*-lytic mode. The elucidation of TT16 will benefit the preparation of GAG disaccharides and the improvement of enzyme properties through rational design and gene mutation. It will also provide a new potential tool to learn the inner structure–function relationship of GAG carbohydrates.

## 2 Materials and methods

### 2.1 Isolation, identification, and culture of bacterial strains

Coastal sediments were collected from Jiaozhou Bay (China) in 2015, from which bacterial strains were isolated using modified Tryptic Soy Broth (TSB) that contained 3.0% NaCl (w/v), cultured at 28 °C. Morphological observations were performed using optical microscopes.

To determine the bacterial growth capabilities by polysaccharide utilization, each strain was initially grown on the sole-carbon source media containing the following polysaccharides, that is, agarose, alginate, cellulose, microcrystalline cellulose, chitin, chitosan, chondroitin sulfates (CS-A, C, D, and E types), HA, HS/DS mixture, xanthan, and xylan, which were purchased from Sigma–Aldrich Co. Ltd., USA. The medium for screening was composed of 3.0% NaCl, 0.75% KCl, 0.11% CaCl_2_, 0.72% MgSO_4_, 0.15% NH_4_Cl, and 0.10% of each carbohydrate, with a pH of 7.0. After the bacterial culture for more than 24 h, measurements of the cell density (*A*_600_) were individually performed. Negative controls were performed using polysaccharide-free media. Furthermore, to find bacterial strains with polysaccharide-degrading capabilities, every strain was initially cultured for 72 h in the modified TSB broth. The extracellular enzyme was then prepared using the ammonium sulfate precipitation method. After being dialyzed against buffer A [50 mM Tris, 150 mM NaCl, 5 mM disodium salt of ethylenediaminetetraacetic acid (Na_2_EDTA), pH 8.0] to remove and exchange salts, the extracellular enzyme preparation was reacted with every testing polysaccharide substrate at 50 °C for 1 h. The reaction system was finally determined to be active by the 3,5-dinitrosalicylic acid (DNS)-reducing sugar method (Miller, 1959). Control groups were performed using enzyme-free buffer A.

For molecular identification, the bacterial genomic DNA was initially extracted using the Fast Genomic DNA Extract kit (Novate, Nanjing, China) according to the protocol, and then used as a template in the polymerase chain reaction (PCR) of bacterial 16S rRNA gene, using the universal primers 27F (5′-AGAGTTTGATCCTGGC TCAG-3′) and 1492R (5′-TACGGCTACCTTGTTACGACTT-3′).

PCR products were then gel recovered and verified by agarose electrophoresis. After being ligated into the pEasy-Blunt Simple Cloning Vector (TaKaRa, Dalian, China), the resultant plasmids were transformed into *Escherichia coli* DH5α cells. Positive clones were obtained by screening for ampicillin resistance and were then sent to a biological company for DNA sequencing. The resultant 16S rRNA gene sequence was analyzed using the Nucleotide Basic Local Alignment Search Tool (BLASTn) and Global Alignment searches against the database of standard strains at the National Center for Biotechnology Information (NCBI) website^2^ (Schmidt et al., 1991).

Cells of *E. coli* strains, that is, DH5α or BL21 (DE3), were cultured at 37°C for gene cloning or at 16°C for protein production in Luria-Bertani (LB) broth, respectively, supplemented with ampicillin (100 μg/ml) or kanamycin (50 μg/ml) when necessary. Solid medium plates were prepared by adding additional agar powder (1.5%, w/v).

### 2.2 Data mining and gene analysis

To obtain the genome sequence for further data mining, the genomic DNA of the polysaccharide-degrading strain WS15 was initially applied for library construction, which was then sequenced using the pyrophosphate sequencing technology. Furthermore, the genome sequence was PCR-verified and data-assembled by Meiji Biotech Co. Ltd. Finally, it was annotated using the program integrated into the NCBI website (Burgoon and Zacharewski, 2008), for example, BioProject. To screen the encoding genes of candidate GAG lyases, putative proteins were analyzed as follows:

DNA sequence of the candidate gene *tt*16, encoded by the WS15 genome, was translated into the amino acid sequence of the predicted protein TT16, using BioEdit software version 7.02 (Hall, 2011). The signal peptide was identified using the SignalP online server 5.0^3^ (Almagro Armenteros et al., 2019). The molecular weight and the isoelectric point (pI) were estimated using the peptide mass tool on the ExPASy server of the Swiss Institute of Bioinformatics.^4^ Based on the CAZy database (see footnote 1) and the SMART online server,^5^ sequence similarity searches of the TT16 protein to characterized enzymes were performed using the BLASTp algorithm on the NCBI server. Multiple sequence alignments and phylogenetic analyses were then performed using MEGA software version 7.2.5 (Kumar et al., 2016).

### 2.3 Gene cloning

To construct a recombinant plasmid, initially the gene *tt*16 was amplified from the genomic DNA of strain WS15, using the high fidelity Vazyme™ LAmp DNA Polymerase (Vazyme, Nanjing, China) and the primers rCTF-TT16-F (5′-CATATGTCGCTGCAGCCGCT GAGC-3′) and rCTF-TT16-R (5′-TCTAGAGCGGTGCAGCGAG AACTCCAG-3′), which were designed for the restriction enzyme sites (underlined) of *Nde* I and *Xba* I (TakaRa, Dalian, China). The resultant PCR product was then gel-recovered, enzyme-digested, and finally ligated into the vector pCold TF™ by T4 DNA ligase, producing the plasmid pCTF-TT16 to yield rCTF-TT16, one recombinant protein of TT16, which was fused with a Trigger Factor (TF) for dissolution enhancement at the *N*-terminus and a 6 × His tag for affinity to Ni^2+^ at the *C*-terminus, respectively. DNA was sequenced for fidelity.

Moreover, the gene *tt*16 was initially PCR-amplified using the primers rCTF-TT16-F and rTT16-R (5′-CTCGAGGCGGTGCAGC GAGAACTCCAG-3′), which were designed for *Nde* I and *Xho* I, and finally cloned into the plasmid pET30a (+) ™ (Invitrogen, USA). The resultant plasmid pET30-TT16 was applied to produce rTT16, the other recombinant protein of TT16, which was fused with a 6 × His tag at the *C*-terminus.

### 2.4 Protein expression and purification

For protein expression, *E. coli* BL21 (DE3) cells harboring the plasmid pCTF-TT16 or pET30TT16 were initially cultured in 100 ml LB broth individually, and shaken for approximately 3 h at 200 rpm till the cell density reached an *A*_600_ value of 0.7 ~ 0.8. Cells were then induced to express target proteins by supplementing with isopropyl 1-thio-*β*-D-thiogalactopyranoside (IPTG) at a final concentration of 0.01–0.50 mmol, and shaken at 220 rpm for another 8 to 24 h. Cells were harvested by centrifugation at 10,000 *g*, 4 °C for 10 min, washed and resuspended using ice-cold buffer B (50 mmol/L Tris, 150 mmol/L NaCl, pH 8.0), and disrupted by sonication (80 repetitions, 5 s). After a centrifugation at 15,000 *g*, 4 °C for 30 min, the supernatant containing soluble recombinant proteins was loaded onto a buffer B-equilibrated Ni-nitrilotriacetic acid agarose (NiNTA) column (TaKaRa, Dalian, China). The protein-bound column was eluted using buffer B, which contained imidazole in gradient concentrations: 0, 10, 20, 50, and 250 mmol/L. Fractionated samples were analyzed using sodium dodecyl sulfate–polyacrylamide gel electrophoresis (SDS–PAGE) for protein assembly. Finally, to obtain active enzyme preparations, purified protein fractions were dialyzed against buffer C (50 mmol/L Tris, 50 mmol/L NaCl, 5.0 mmol/L Na_2_EDTA, 5.0% glycerol (v/v), pH 8.0).

SDS–PAGE was performed using 12.0% (w/v) polyacrylamide gels according to the method of Sambrook and Russell (Sambrook and Russell, 2001). Proteins were detected by staining gels with Coomassie Brilliant Blue R-250. Protein concentrations were individually determined by the Folin–Lowry method using Folin–Ciocalteu’s phenol reagent (Sigma–Aldrich, USA), with bovine serum albumin as standard (Sambrook and Russell, 2001).

### 2.5 Enzyme activity and biochemical characterization

Initially, to determine the substrate spectrum, each listed polysaccharide was dissolved in deionized water to prepare a stock solution (3.0 mg/ml) for substrate tests. Each stock solution (100 μl) was then mixed with 70 μl water, 30 μl of appropriately diluted enzyme (0.10–10 U/ml) of rCTFTT16 or rTT16, and 100 μl buffer (50 mmol/L NaAc-HAc, pH 6.0), and mixed and incubated at 50 °C. Enzyme-treated samples were heated in boiling water for 10 min and then cooled with ice. After a centrifugation at 15,000 *g*, 4 °C for 10 min, the product-containing supernatant was finally collected and analyzed by measuring the absorbance at 540 nm using the DNS-reducing sugar method ^(38)^. One unit was defined as the amount of enzyme required to release 1 μmol of the reducing sugars per minute under the optimal reaction conditions.

Next, the optimal temperature was determined by using the HA, CS-A, CS-C, CS-D, and CS-E polysaccharides as a testing substrate individually, in a 50 mmol/L NaAc-HAc buffer (pH 6.0) at temperatures ranging from 0 to 70 °C for 1 h. To determine the thermostability of rTT16, residual enzyme activities were measured by pre-incubating the enzyme at various temperatures, ranging from 0 to 70 °C, for time-course intervals of 0 to 72 h. The optimal pH value was determined by using the following buffers with different pH values: 50 mmol/L NaAc-HAc buffer (pH 5.0–6.0), 50 mmol/L NaH_2_PO_4_–NaHPO_4_ buffer (pH 6.0–8.0), or 50 mmol/L Tris–HCl buffer (pH 7.0–10.0, adjusted at 4 °C). Effects of different pH values on the enzyme stability were determined by measuring the residual activities after incubating each recombinant enzyme at 4 °C at various pH values (5.010.0) for 2 h. Effects of metal ions and chelating agents on the polysaccharide-degrading activity were examined by determining the relative activity of each reaction in the presence of 1 or 10 mmol/L of associated chemicals. All reactions were performed in triplicate. Negative controls were performed without associated chemicals, and their residual activity was assigned a value of 100%.

The enzyme kinetics of reverse transcriptase of the F plasmid (rTF)-TT16, for example, *K*m and *V*max values, have been tested and determined recently, using the standard Lineweaver–Burk plots as described (Sun et al., 2019).

### 2.6 High performance liquid chromatography (HPLC) analyses

Every polysaccharide substrate, that is, HA, CS-A, CS-C, CS-D, and CS-E, was initially dissolved in water to prepare stock solutions (3.0 mg/ml), and then digested using the methods previously described for enzyme activity tests, for example, with the final enzyme concentration of rTT16 or rCTF-TT16 (0.10–10 U/ml), in the 50 mmol/L NaAc-HAC buffer (pH 6.0) at 50 °C, and for a time course ranging from 0 to 72 h. Subsequently, 1.0 μg of reacted mixture (approximately 20 μl) was injected and observed at 232 nm via gel filtration HPLC by a Superdex™ 30 Increase 10/300 GL GE HealthCare (GE) column, using 0.20 mol/L NH_4_HCO_3_ at a flow rate of 0.40 ml/min. Finally, fractionized oligosaccharide products were frozen and repeatedly dried to remove water and the NH_4_HCO_3_ salt for further NMR tests. All reactions were performed in triplicate. Negative enzymatic controls were performed with inactive enzymes or enzyme-free buffers only.

### 2.7 Product structure identification

Chemical structures of the resultant final oligosaccharide product fractions were further determined, for example, approximately 3.0 μg final oligosaccharides were tested using the tandem mass spectrometry (the Quadrupole Time-of-Flight Mass Spectrometer) and approximately 1.0 mg final products were injected into ^1^H-NMR spectroscopy with a JNM-ECP600 (JEOL, Japan) instrument set at 600 MHz, respectively.

### 2.8 Protein modeling and molecular docking

The three-dimensional protein structure of TT16 was initially calculated and homologically constructed on the Swiss-model website (see footnote 4) (Zhang et al., 2012), with the crystal structure of chondroitin AC lyase [Protein Data Bank (PDB) number: 1rw9] (Féthière et al., 1999) as a template. The resultant protein model.

(acceptor) was then molecule-docked with the substrate (donor) of HA, CS-A, CS-C. CS-D, and CSE tetrasaccharide using the software AutoDock Vina 1.2.5 (Bugnon et al., 2024) and through the PLIP online analysis^6^ (Schake et al., 2025). The binding energy of each of the most stable enzyme-oligosaccharide complexes was calculated and visualized using the PyMOL 2.1.1 software (Rauf et al., 2015) and LigPlot^+^ software (Laskowski and Mark, 2011). Key active site residues and their roles were estimated according to reported references (Elmabrouk et al., 2011; Zou et al., 2025).

## 3 Results

### 3.1 Isolation and identification of the WS15 strain

A bacterial strain, namely, WS15, was isolated from the coastal sediment of Jiaozhou Bay that was collected in 2015. The bacterium was positive in Gram-staining, and showed yellow opaque colonies on 3.0% NaCl-containing TSB solid media. Cells in fresh media (cultured for less than 30 h) were elongated, irregular bacilli, singly or in pairs, and partially arranged at right angles to V shape; however, in aged cultures, they were rod-shaped and spheroidal, without an obvious rod-sphere cycle.

**Figure 1:**
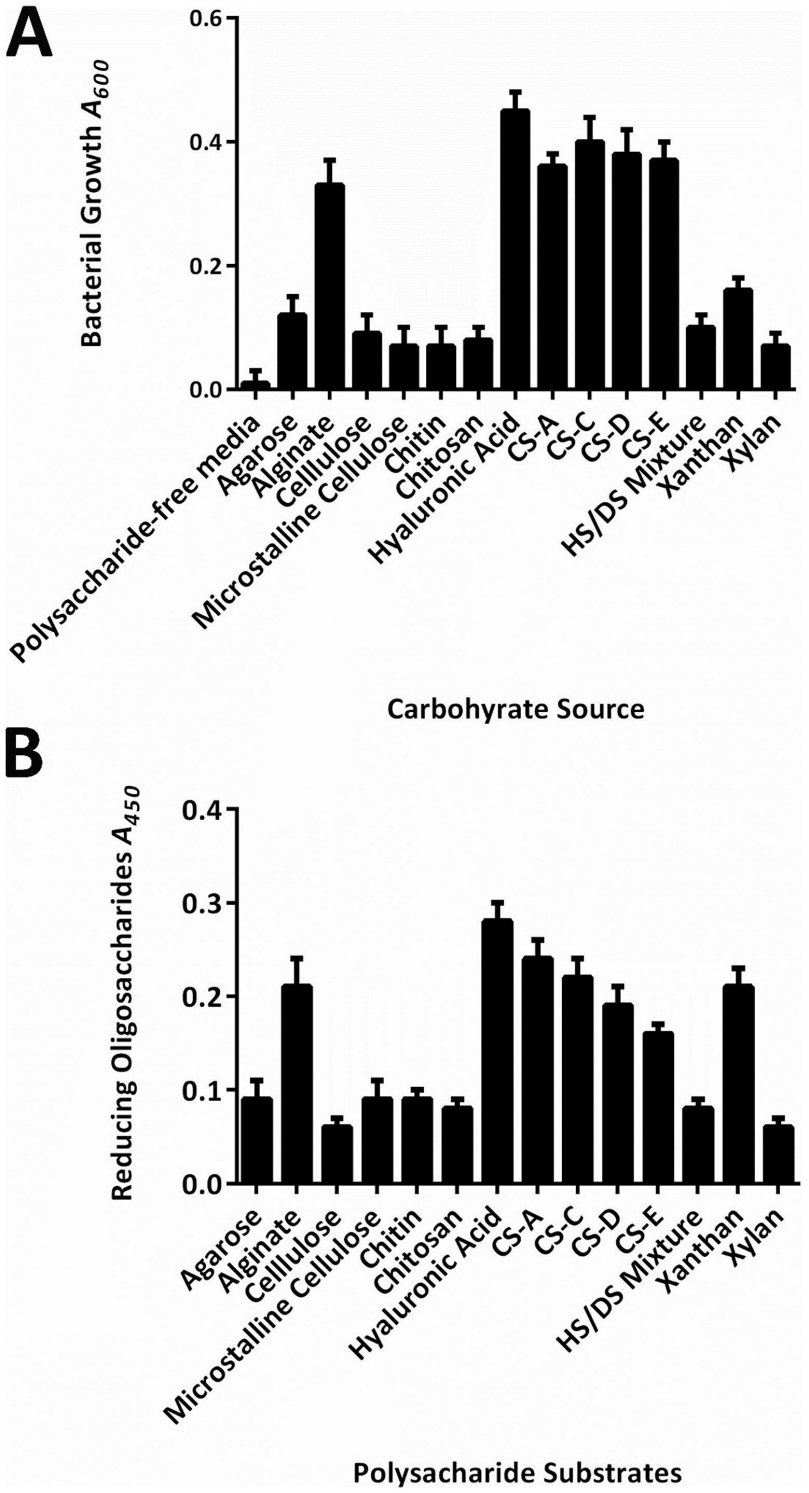
Carbohydrate utilization of the strain WS15. **(A)** Cell densities (*A*_600_) of growth on different sole-carbon source media. **(B)** DNS-reducing sugar (*A*_450_) of the enzymatic digestion against various polysaccharides.

Moreover, strain WS15 could hardly grow on agarose, cellulose, and chitin, whereas it could grow efficiently on six kinds of polysaccharides as the sole-carbon source (Figure 1A), especially with the *A*_600_ values of cell densities greater than 0.20 to show obvious turbidity when grown on alginate, HA, and four tested CS polysaccharide types, that is, CS-A, CS-C, CS-D, and CS-E. Furthermore, by DNS-reducing sugar tests, the bacterial extracellular enzyme preparation could degrade nearly little agarose, cellulose, and chitin to produce significant oligosaccharide products, while it could degrade at least seven types of polysaccharides to yield reducing oligosaccharide products for further bacterial utilization, in particular with the *A*_450_ values of reducing sugar product greater than 0.20 when against alginate, HA, and xanthan (Figure 1B).

By gene cloning and sequencing, the 16S rRNA gene of the bacterium WS15 (CGMCC No. 13,421) was 1,447 base pairs (bp) in length, and deposited under the No. PQ312688 to GenBank. By performing the BLASTn program combined in the NCBI website, the gene of strain WS15 showed high sequence identities to those of standard strains, with the highest to *Microbacterium esteraromaticum* strain ATCC 8091 (99.8%), and *M. arabinogalactanolyticum* strain ATCC 51926 (99.3%), respectively (Takeuchi and Hatano, 1998).

Therefore, the marine-derived strain WS15 is defined as a polysaccharide-degrading bacterium, belonging to the *Microbacterium* genus of actinomycetes.

### 3.2 Sequence property of TT16

**Figure 2:**
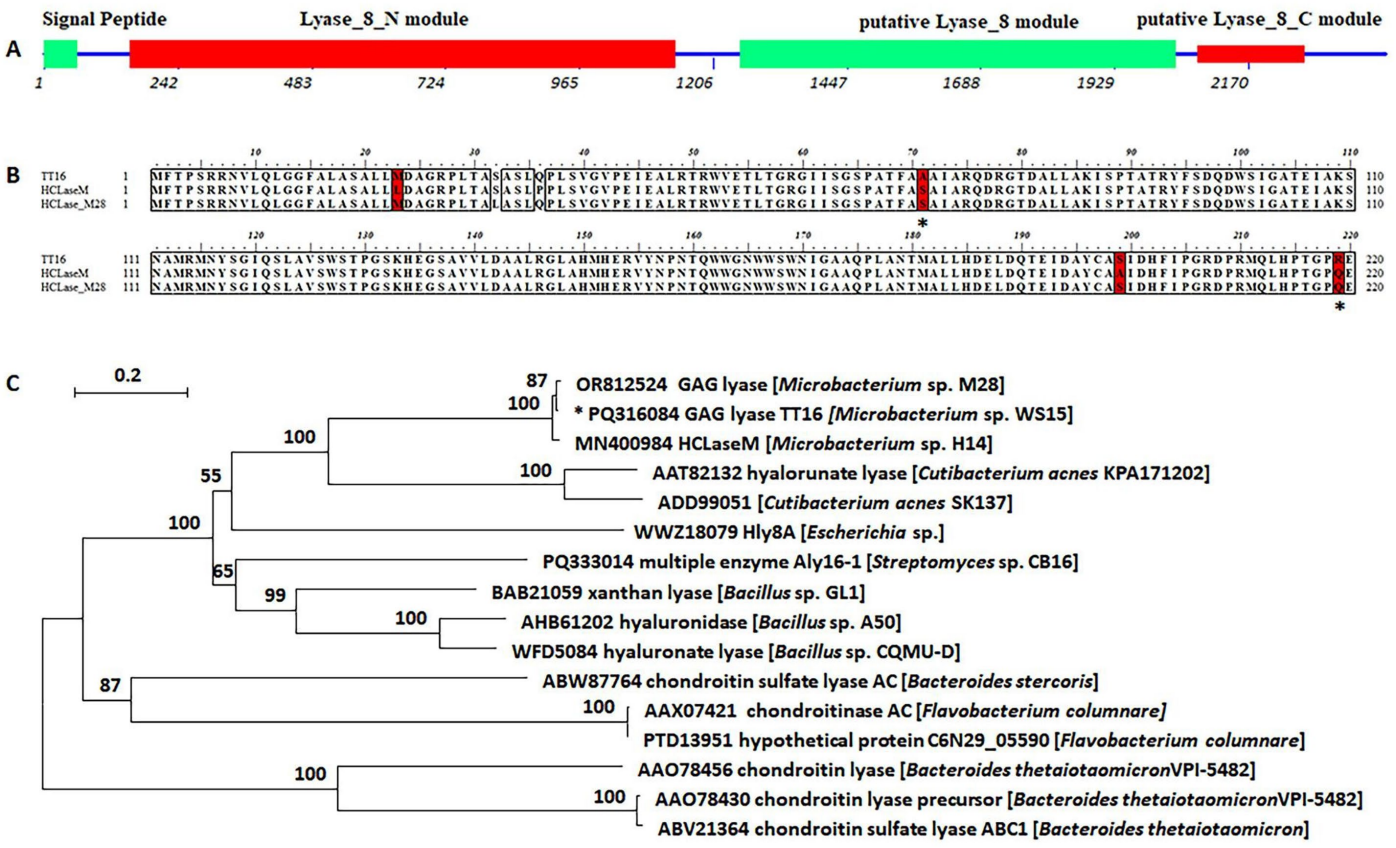
Sequence property of the protein TT16. **(A)** Modular architecture of the protein TT16 and the numbers indicated corresponding nucleotides of the encoding gene. **(B)** Partial sequence alignment of the *exo*-lytic enzyme TT16 and elucidated *endo*-lytic lyases of HCLaseM and HCLase_M28, and candidate amino acid residues corresponding to the action type were indicated by “*.” **(C)** Phylogenetic analysis based on the protein sequence alignment. The neighbor-joining tree was obtained using MEGA version 7.05 software. The numbers on the branches indicate the bootstrap confidence values from 1,000 replicates. The bar is equal to the distance corresponding to two amino acid substitutions per 10 amino acid residues.

The genome of strain WS15 contained a gene *tt*16, which was 2,415 bp in full length and registered under GenBank No. PQ316084. The gene encoded a putative polysaccharide lyase (TT16) of 804 amino acids, with a molecular mass of approximately 85.9 kDa, an isoelectric point (pI) of 6.1, and a type I signal peptide at the *N*-terminus (Met^1^ to Ala^20^) (Figure 2A). SMART online analysis of the protein TT16 indicated the domain composition and organization, including a putative *N*-terminal module (Trp^52^ to Gln^379^) of the Lyase_8_N module, followed by a putative Lyase_8 module (Pro^418^ to Asp^679^), as well as a putative Lyase_8_C module (Arg^693−^Val^756^) in the *C*-terminus (Figure 2A) (Elmabrouk et al., 2011; Zou et al., 2025; Rani et al., 2018; Rye and Withers, 2002).

The protein TT16 shared sequence identities of 98 and 99% with two GAG lyases of *Microbacterium* strains, that is, the HCLaseM of *Microbacterium* sp. strain H14 (Sun et al., 2019) and the HCLase_M28 of *Microbacterium* sp. strain M28 (Ju et al., 2024), respectively, which have never been reported as *endo*-lytic lyases. Moreover, they were defined into the same cluster of the PL8 family (Figure 2B). Based on the latest CAZy database (downloaded on 23 June 2025), “Global Alignment” searches on the NCBI website showed that the protein TT16 shared low sequence identities with other characterized PL8 enzymes, that is, less than 30% with 46 microbial hyaluronan lyases or chondroitin sulfate lyases. Notably, a few differences were shown in the sequence alignment, for example, it was the residues Ala71 and Arg 219 in the TT16 sequence, while both were residues Ser71 and Gln219 in the HCLaseM and the HCLase_M28 (Figure 2B), the positions of which aroused interest in the following protein-structure modeling and molecule docking. It showed approximately 36% down to 30% similarities with the HA lyases of the *C. acnes* strain KPA171202 (GenBank AAT82132, 36%), the *C. acnes* strain SK137 (GenBank ADD99051, 34%), and the *Streptomyces coelicolor* strain A3(2) (GenBank CAA19982, 34%), till the multiple enzyme Aly161 of the *Streptomyces* sp. strain CB16 (GenBank PQ333014, 32%) (Figure 2C) (Zeng et al., 2024). Therefore, the protein TT16 is predicted to be a PL8 GAG lyase member, with the substrate spectrum to be determined by experiments.

### 3.3 Protein purification and biochemical characteristics

**Figure 3:**
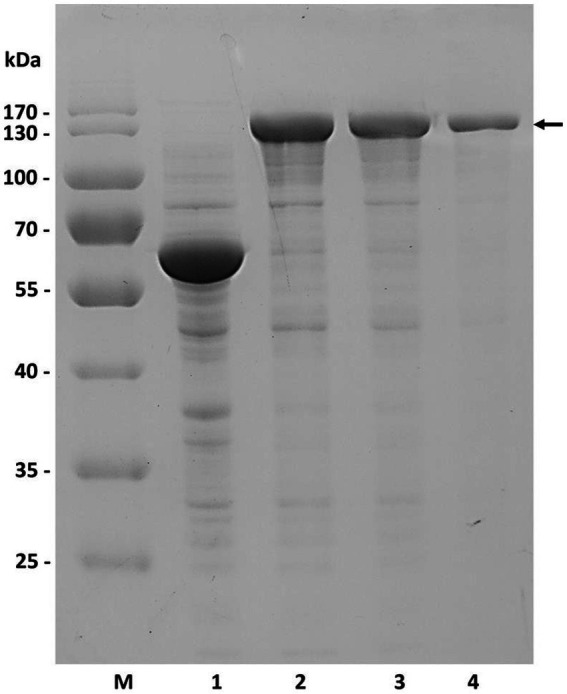
Expression and purification of the recombinant protein rCTF-TT16 (SDS–PAGE). M, protein molecular weight standards with the sizes of 170, 130, 100, 70, 55, 40, 35, and 25 kDa. Lane 1, lysate of the controlling *E. coli* BL21(DE3) cells containing the plasmid pCold TF™. Lane 2, cell lysate of the positive bacteria containing the plasmid pCTF-TT16. Lane 3, supernatant of Lane 2. Lane 4, purified protein of rCTF-TT16.

To construct recombinant vectors, the gene *tt*16 was initially PCR-amplified using primers associated with the corresponding plasmid, with the WS15 genomic DNA as template. After gel recovery, the resultant 2.4 kb DNA fragment was enzyme-digested with either *Nde* I and *Xba* I or *Nde* I and *Xho* I, and then ligated into enzyme-digested pCold TF™ (TaKaRa, Dalian, China) or pET30 (+) ™ (Invitrogen, USA) by the T4-DNA ligase, yielding the recombinant plasmids pCTFTT16 and pET30-TT16, respectively. The resultant fusion proteins, rCTF-TT16 or rTT16, were each expressed in recombinant *E. coli* strain BL21(DE3) cells, induced at a cell density (*A*_600_) of approximately 0.7, with IPTG at a final concentration of 0.05 mmol/ml. Subsequently, induced cells were disrupted by sonication, and the target protein rCTF-TT16 was purified by affinity chromatography from sonicated cell fractions, using agarose-derived Ni^2+^-affinity gels with imidazole at concentrations greater than 50 mmol/ml. As a result, a protein band, consistent with the predicted molecular weight, was shown in a 12.0% SDS–PAGE gel (Figure 3). The active enzyme preparation was finally obtained through dialysis against 50 volume folds of buffer C, four times at 4 °C.

**Figure 4:**
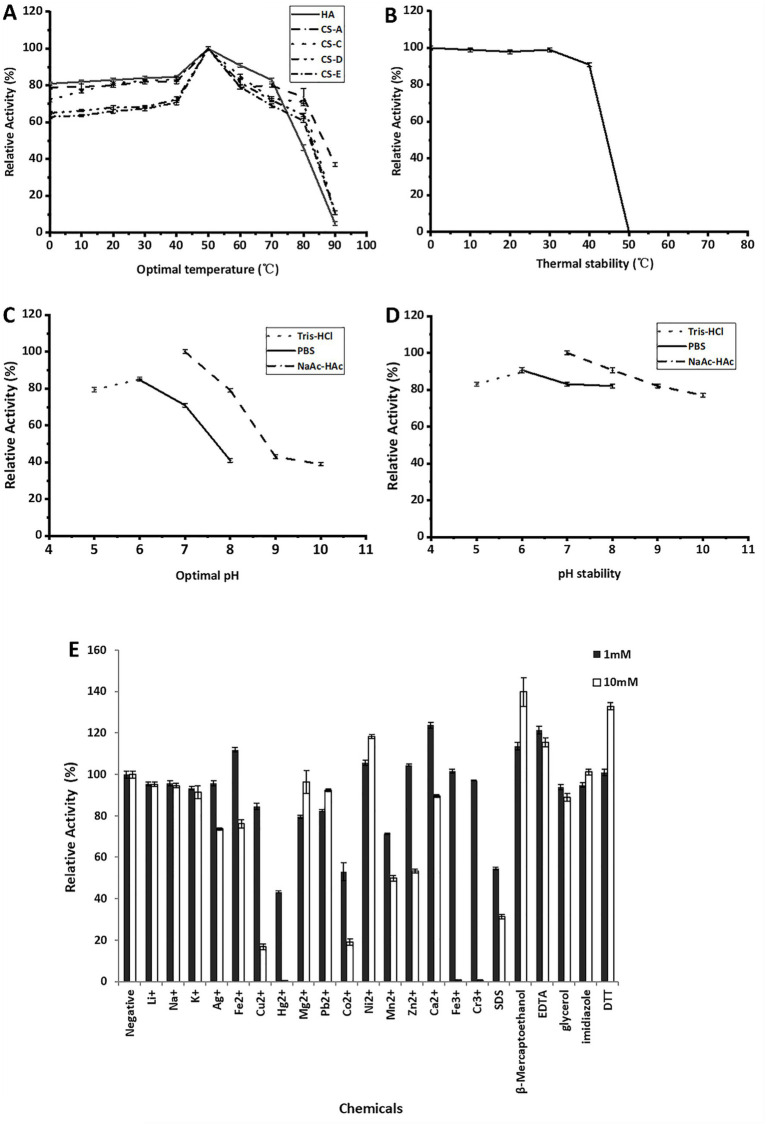
Biochemical characteristics of the enzyme rCTF-TT16. **(A)** The optimal temperature against various polysaccharide substrates. **(B)** Thermal stability of rCTF-TT16 on various polysaccharides. **(C)** The optimal pH value against the HA polysaccharide. **(D)** pH stability against HA. **(E)** Effects of different metal ions and chemicals on the activity of rCTF-TT16 in the presence of 1 and 10 mmol/L, respectively. Average values ± standard bars were indicated.

In DNS-reducing sugar tests, the enzymes rCTF-TT16 and rTT16 showed similar biocharacteristics to each other. The enzyme always showed the highest activity at 50 °C, whenever being reacted with each testing substrate of GAG polysaccharide, that is, HA, CS-A, CS-C, CS-D, or CS-E (Figure 4A). It retained more than 50% residual activity after pre-incubation at temperatures from 0 to 40 °C for 6 h (Figure 4B). Moreover, the enzymes were active even when reacted or preincubated under low temperatures, for example, 0 °C (Figures 4A,B), implying cold-adaptive characteristics (Sarmiento et al., 2015; Liu et al., 2023). With the HA polysaccharide as substrate, the optimal pH value of rTT16, determined at 50 °C in 50 mmol/L NaAc-HAc buffer, was 6.0 (Figure 4C). The enzyme remained stable, showing residual activity ≥ 80% in each testing environment (pH 6.0–10) after pre-incubation at 4 °C for 2 h (Figure 4D). As shown in Figure 4E, at the concentration of 1 or 10 mmol/L: (1) Na^+^, K^+^, Li^+^, and Ag^+^ had a weak inhibitory effect on the rTT16 activity at 1 mM, while Ag^+^ had a significant inhibitory effect at 10 mmol/L. (2) Fe^2+^, Ni^2+^, Ca^2+^, and other divalent metal ions could promote the enzyme activity, while other divalent and trivalent metal ions were able to inhibit the enzyme activity. (3) *β*-mercaptoethanol, EDTA, and DTT could significantly promote the activity at the 10 mmol/L concentration, increasing the enzyme activity to 139.8, 115.5 and 132.9%, respectively.

### 3.4 Substrate spectrum, catalytic properties, and action modes

To investigate the substrate spectrum and determine the substrate preference of TT16, a total of 14 types of polysaccharides were individually used as testing substrates in enzyme reactions with rCTF-TT16 or rTT16 for DNS-reducing sugar tests. After reactions for 72 h, the product mixture of each polysaccharide substrate was examined through gel filtration HPLC analysis, monitored at 232 nm. As a result, five polysaccharide types—HA, CS-A, CS-C, CS-D, and CS-E—could be digested by the enzymes to yield obvious reducing sugars in the DNS tests, indicating a broad substrate spectrum of TT16 against GAGs. Furthermore, the enzyme rTT16 showed the highest activity (1,840 U/mg) against the HA polysaccharide, followed by the activities against the CS-A (1,620 U/mg) and CS-C (1,550 U/mg) polysaccharides, and the smallest activities against the CS-D (1,510 U/mg) or the CS-E (1,570 U/mg) polysaccharide substrates, implying an activity increase contrary to the complexity of sulfation in GAG substrates. The protein concentration of rTF-TT16 was approximately 0.5–1.5 mg/ml for testing and determining kinetics. If compared to those *K*m values of reported *endo*-type GAG lyases, for example, HCLaseV (Wang et al., 2022) (0.919 mg/ml) and HCLaseM (Sun et al., 2019) (0.419 mg/ml), the smaller value of rTF-TT16 (0.088 mg/ml) indicated a higher efficiency for GAGs degradation (Supplementary Figure S1A). The recombinant enzymes of TT16 showed the most preference to polysaccharide substrate HA, followed by CS-A, CS-C, and CS-D; finally, CS-E (Supplementary Figure S1A,B).

**Figure 5:**
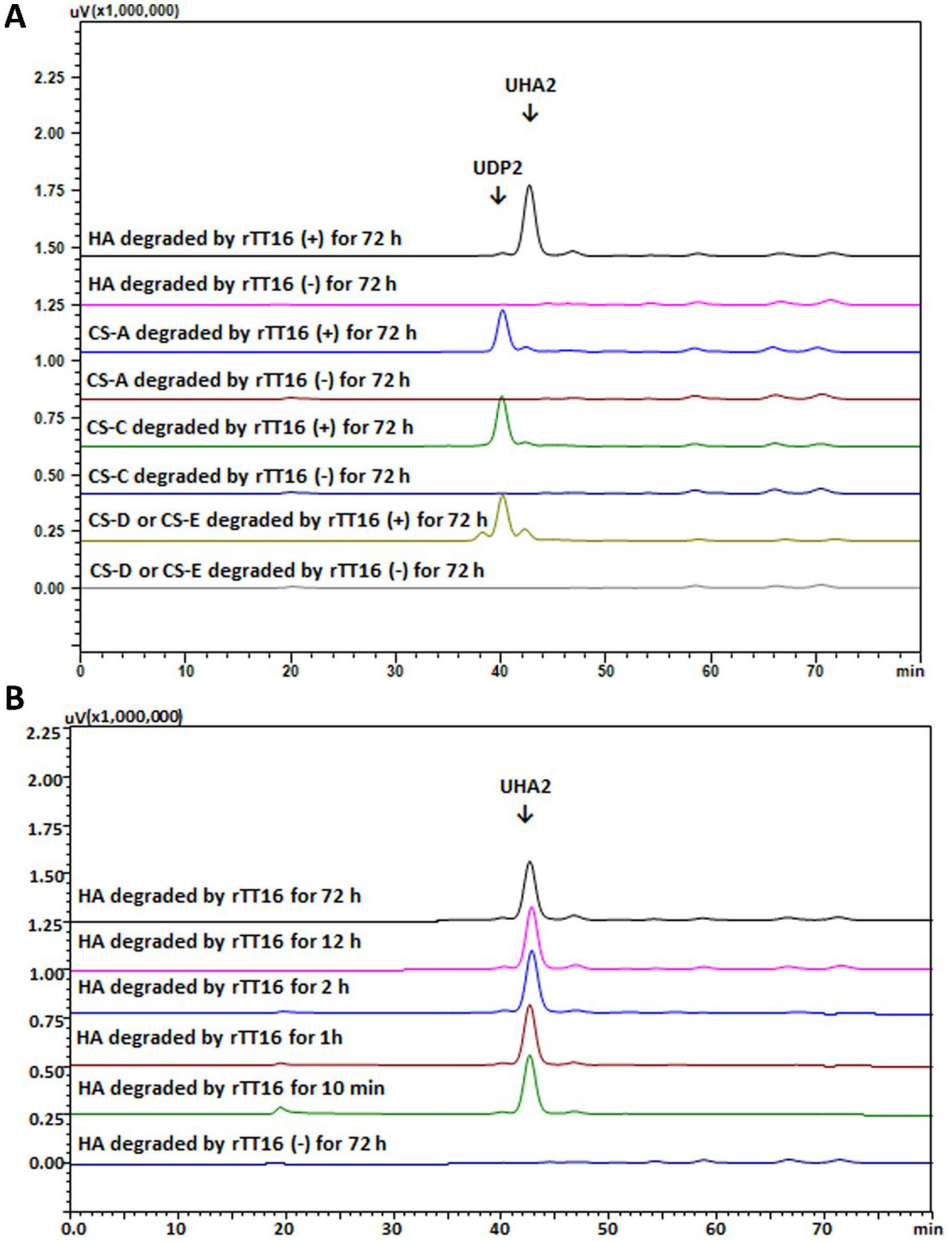
Gel filtration HPLC analysis of the GAG degradation by TT16 using the GAG substrates and the Superdex™ peptide 10/300 column. **(A)** The degradation modes of rTT16 against HA and various CS polysaccharides, yielding unsaturated CS disaccharide (UDP2) or unsaturated HA disaccharide (UHA2); **(B)** Time-course HPLC analysis of oligosaccharide products during the digestion of HA by rTT16; Negative group (−): reactions without any enzymes.

As shown in Figure 5A, when reacted with rCTF-TT16 or rTT16, five GAG polysaccharide substrates—HA, CS-A, CS-C, CS-D, and CS-E types—could yield unsaturated disaccharide products (UDP2), according to their absorption at 232 nm and corresponding retention time in gel filtration HPLC analyses. Notably, unsaturated disaccharide products (UHA2) were yielded from the start to the end of the HA digestion (Figure 5B), indicating a typical *exo*-type mode as reported (Kanda et al., 1989). Interestingly, when the CS-E polysaccharide was digested, peaks of the unsaturated disaccharide products (UDP2) were yielded along with a smaller proportion of two noisy peaks (Figure 5A), which might be UDP2 that are sulfate-free or that are modified with three sulfate groups, according to the retention time comparison. Therefore, the protein TT16 has a catalytic property of disaccharide-yielding in the *exo*-lytic mode.

**Figure 6:**
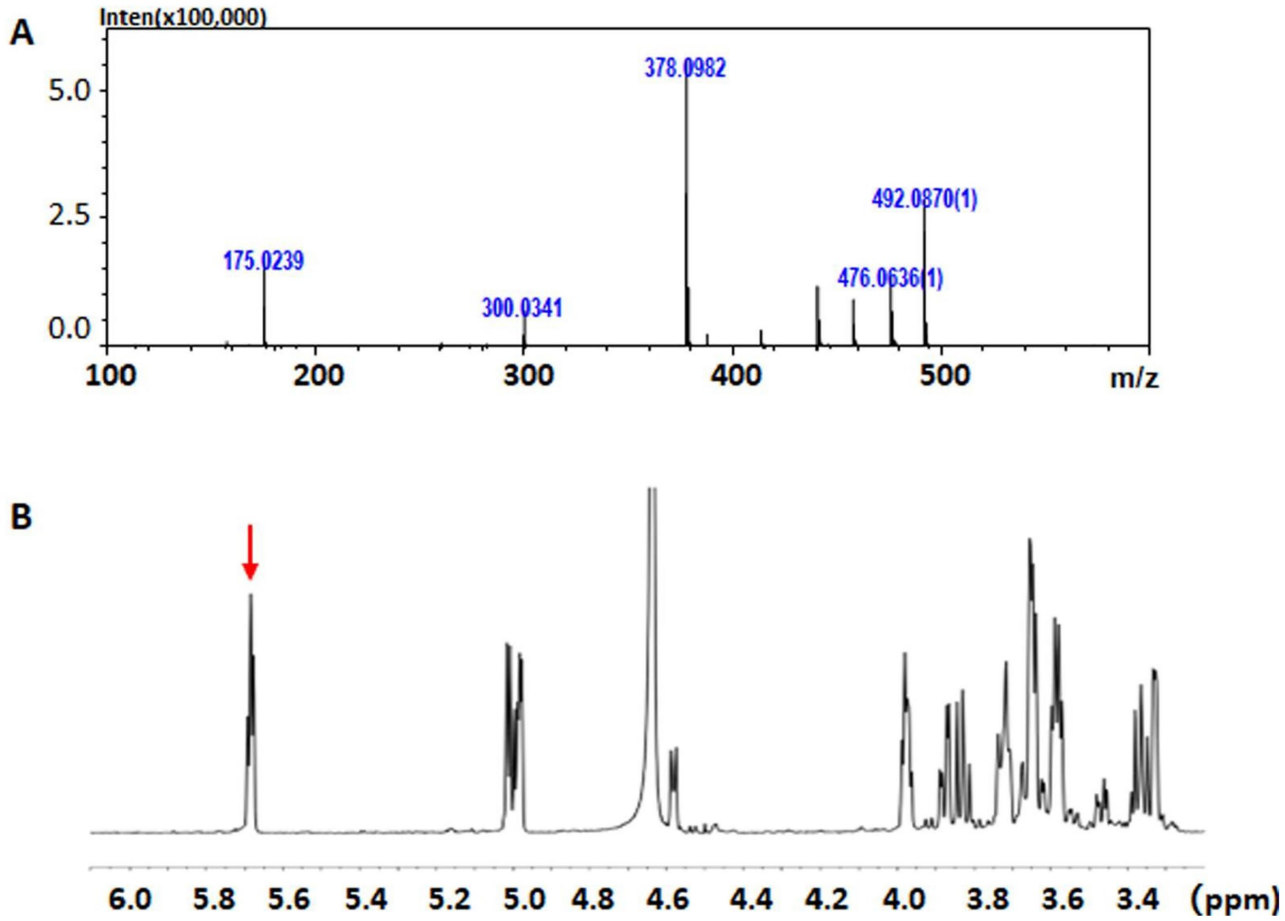
The mass spectrum **(A)** and the ^1^H-NMR spectrum **(B)** of HA-derived unsaturated disaccharide products. **(A)** The m/z peak of 378 indicates a pseudo-anion [379-H]^−^, suggesting an unsaturated HA disaccharide. **(B)** The signals of H-absorbance at ~5.7 ppm were indicated by a red arrow, meaning the β-elimination of a GAG lyase.

To further identify the structures of the final oligosaccharide products, approximately 10 mg HA-derived end digestions were initially fractionated through gel filtration HPLC. These were the collected as disaccharide fractions, which were finally dried and freeze-dried repeatedly. As shown in Figure 6A, the HA-derived disaccharide product showed a m/z peak of 378, indicating the [mH]^−^ pseudo-ion in the primary anion mass spectrum, implying the yield of unsaturated HA disaccharides (UHA2, with a mass of 379) by rTT16. Furthermore, when compared to the control groups, specific signals of H-absorbance at ~ 5.7 ppm were significantly enhanced in the HA-derived end digestions (Figure 6B), indicating a conjugated structure produced by the *β*-elimination of the enzyme rTT16. Therefore, these results indicated that the enzyme TT16 can digest multiple acidic polysaccharides, i.e., many GAG types including HA, CS-A, CS-C, CS-D, and CS-E, via the *β*-elimination mechanism as a lyase.

## 4 Discussion

In this study, a new bacterium strain WS15 was initially isolated from coastal sediments using artificial TSB medium, subsequently determined to have capabilities of utilizing a wide of polysaccharides from animals, microorganisms, or plants for bacterial growth (Figure 1A), and finally identified to be a strain within the *Microbacterium* genus, an actinomycete, by the 16*S* rRNA gene. Notably, the marine-derived bacterium was capable of growing well on multiple GAG polysaccharides, for example, HA and many CS types, probably due to the primary digestion by extracellular enzymes (Figure 1B) and further bacterial utilization (Figure 1A), ultimately for the GAG-degrading systems (Rawat et al., 2022a; Shain et al., 1996), meaning a new resource bacterium to be explored.

By further genome sequencing and data mining, the encoding gene of a potential GAG lyase TT16 was obtained from the WS15 genome. Based on protein analyses of the sequence similarity, the molecular organization (Figure 2A), and the evolutionary property (Figure 2B), the protein TT16 was predicted to be a new PL8 family member, while with the substrate spectrum and the optimal substrate yet to be determined by experiment. Thus, the encoding gene *tt*16 was PCR-amplified and enzyme-cloned to construct recombinant plasmids, which were then induced by IPTG to express fusion proteins. After being purified by affinity chromatography with imidazole, a recombinant protein rCTF-TT16 was successfully obtained (Figure 3) and dialyzed against buffer C to obtain the active enzyme preparation for further tests.

In general, elucidated lyases containing GAG modules could broadly degrade various GAG polysaccharide substrates (Elmabrouk et al., 2011; Zou et al., 2025; Wayne and Sheryl, 2000; Sun et al., 2019; Ju et al., 2024), while few were active against other polysaccharide types except for the multiple enzyme Aly16 − 1 of another actinomycete, *Streptomyces* sp. strain CB16, which could degrade alginate optimally instead (Zeng et al., 2024). Similar to Aly16-1, the protein TT16 contained PL8 modules in sequence (Figure 2A) and shared sequence identities ranging from 30 to 36% with most characterized PL8 family members (Figure 2C) from either marine or terrestrial environments. Moreover, the protein TT16 showed a sequence identity of approximately 98% to the HCLaseM of *Microbacterium* sp. strain H14 (Sun et al., 2019) and approximately 99% to the HCLase_M28 of *Microbacterium* sp. strain M28 (Ju et al., 2024), with only a few different amino acid residues, for example, it was the residues Ala71 and Arg 219 in the TT16 sequence, while both were the residues Ser71 and Gln219 in the HCLaseM and the HCLase_M28 (Figure 2B). Therefore, the protein TT16 would be identified as an *endo*-type GAG lyase according to existing reports. However, the results of detailed experiments were found to be opposite, as follows:

In DNS-reducing sugar tests, both the recombinant enzymes rCTF-TT16 and rTT16 exhibited broad spectra, including multiple GAG polysaccharides. However, quite different from Aly16-1 (Zeng et al., 2024), neither of the two recombinant enzymes could digest alginate to yield reducing oligosaccharides, although the proteins TT16 and Aly16-1 shared a sequence identity of 34% with each other. Therefore, the substrate spectrum of a new PL8 member can be roughly estimated to be broad by bioinformatic predictions, whereas exact experimental tests are needed for a detailed determination.

Interestingly, regardless of whether HA, CS-A, CS-C, CS-D, and CS-E polysaccharide was applied as a substrate, the recombinant enzyme rCTF-TT16 or rTT16 remained optimal at 50 °C and pH 6.0 (Figures 4A,C), indicating no obvious effects of substrate type on biochemical characteristics of enzymes. Furthermore, the biochemical characteristics were significantly different from those reported for HCLaseM (optimal at 35 °C and pH 7.0) (Sun et al., 2019), while similar to those of HCLase_M28 (optimal 50 °C and pH 7.2) (Ju et al., 2024). Notably, the enzymes retained the same biochemical characteristics even when reacted with an excess enzyme concentration of 0.1–10 U/ml or reacted for as long as 96 h. Moreover, these enzymes were cold-adaptive (Figures 4A,C), suggesting the potential application in industrial preparation of unsaturated GAG disaccharides (Sun et al., 2019; Sarmiento et al., 2015; Liu et al., 2023). To further discover the polysaccharide-degrading modes and corresponding oligosaccharide-yielding properties of TT16, enzymatic reactions with various GAG polysaccharide substrates were initially performed for a direct analysis (Figure 5A) or a time-course analysis (Figure 5B) through gel filtration HPLC. During the digestion of HA, the enzymes showed a typical *exo*-type action mode instead of an *endo*-type action mode (Kanda et al., 1989): unsaturated disaccharide products with ultraviolet absorbance at 232 nm were produced exclusively throughout the reaction and were identified primarily according to the retention time (Figure 5B). Furthermore, the sole disaccharide product of HA was molecular-weight confirmed by the mass spectrum (Figure 6A) and finally performed structure identification by the ^1^H-NMR test (Figure 6B) (Guo et al., 2014; Alvarez et al., 2025; Zheng et al., 2024). As a result, the end products were identified as unsaturated disaccharides containing a conjugated structure resulting from *β*-elimination digestion, implying the important potential of TT16 in preparing targeted GAG disaccharides. Thus, the lyase TT16 is quite different from the reported lyases from *Microbacterium* strains, for example, the HCLaseM of *Microbacterium* sp. strain H14 (Sun et al., 2019) and the HCLase_M28 of *Microbacterium* sp. strain M28 (Ju et al., 2024), which could degrade GAG polysaccharides stepwise to produce unsaturated tetra-, hexa-, and even larger size-defined oligosaccharides in *endo*-lytic modes. Notably, the action pattern of TT16 remained unchanged despite variations in substrate types, whether the enzyme concentration was 0.1–10 U/ml or reacted for an excessively long time, for example, 96 h. In a word, TT16 is defined as a broad GAG lyase with the novel catalytic property of *exo*-lytic and disaccharide-producing.

In addition, both the TF-tagged (rTF-TT16) and the tag-free (rTT16) proteins of TT16 were compared, and no significant differences were found between their biochemical characteristic (Figures 4A–E), the initial HPLC analysis of action mode (Figure 5A,B) and the resultant spectral structure identification (Figure 6A,B), even when reacted with an excessive enzyme account or for an excessive long time, indicating that biochemical characteristics, the action mode, and the catalytic property of various enzyme derivative are determined by the TT16 protein instead of the modifying TF tags.Moreover, under optimal conditions, the enzymes of TT16 showed decreased activity against unsulfated HA polysaccharide and various sulfated GAG polysaccharides. Thereby, a seemingly paradoxical conclusion can be drawn: the enzyme TT16 is weakly inhibited by increasing numbers and different positions of sulfation in GAG substrates, indicating a limited resistance to sulfated GAGs; nevertheless, TT16 is tolerant of sulfation and remains active against multiple GAG substrates.

**Figure 7:**
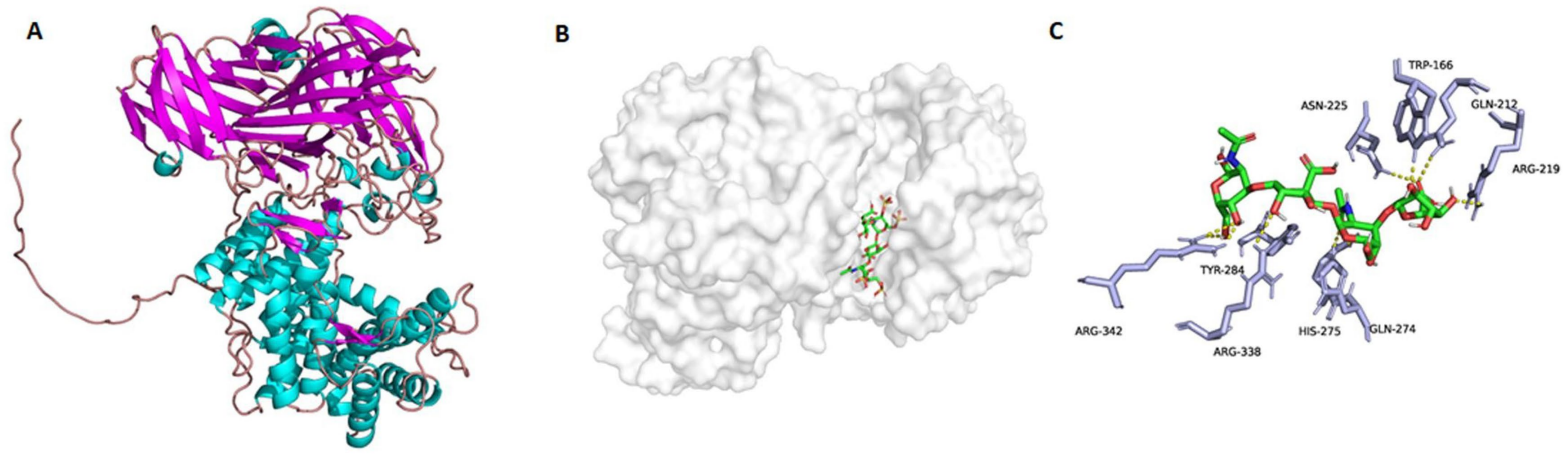
The homology-based protein structure of TT16 **(A)**, he complex with an HA tetrasaccharide substrate **(B)**, and possible bindings to the sulfate-free disaccharide unit **(C)**. The protein TT16 **(A)** combined a tetrasaccharide donor **(C)** with its cavity **(B)** due to the binding and catalysis actions of the key amino acid residues.

**Figure 8:**
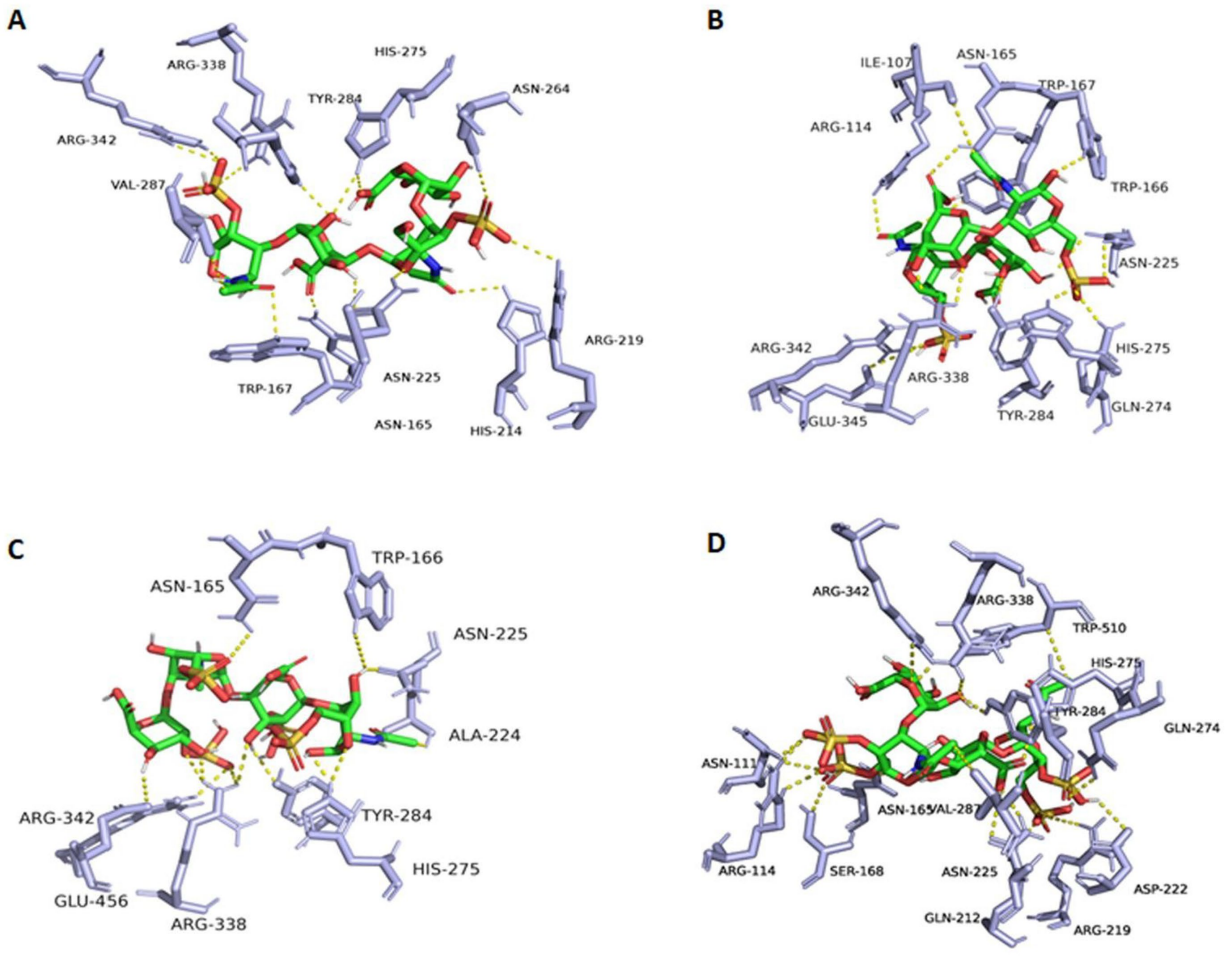
Complexes of TT16 and GAG tetrasaccharide substrates. **(A)** CS-A; **(B)** CS-B; **(C)** CS-D; **(D)** CS-E. By the molecular docking of these various donors to the protein TT16 (acceptor), binding energies of every most stable enzyme-tetrasaccharide complexes were then calculated.

To discover possible bases and mechanisms corresponding to the *exo*-lytic property and action mode of TT16, the three-dimensional protein structure was initially calculated and constructed (Figure 7A) on the Swiss-model website, with chondroitin AC lyase (PDB number: 1rw9) as the homology template. As an analysis result of the software PyMOL 2.1.1, the catalytic cavity of protein TT16 is approximately 20 Å in length. Generally, the size of a chondroitin disaccharide unit is about 10–12 Å (Elmabrouk et al., 2011). Therefore, it is speculated that the enzyme TT16 has a substrate size of at least one HA tetrasaccharide (unsulfated), or two CS units (sulfated). In further molecular docking, the catalytic cavity was flexible enough to accommodate all the HA, CS-A, CS-C, CS-D, and CS-E tetrasaccharide donors successfully (Figures 7B,C). Furthermore, through PLIP^7^ and AutoDock Vina analyses, binding energies of every most stable enzyme-tetrasaccharide complexes were estimated to be −6.910, 6.921, −6.926, −7.092, and −7.514 Kcal/mol individually, which were all smaller than −5.0 *K*cal/mol, and thus being stable (Figures 7C, 8A–D) (Schake et al., 2025). Therefore, the enzyme TT16 provides another protein structure basis capable of strongly binding to each tested GAG-tetrasaccharide substrate for digestion, whether it is unsulfated or sulfated.

Based on the molecular docking, candidate key active site residues of the enzyme TT16 are essential for recognizing, binding, and digesting substrates, which are listed in Table 1. According to previously reported references (Elmabrouk et al., 2011; Zou et al., 2025), their positions within the enzyme, and the binding details to donors (Figures 7C, 8A–D), the residues Arg342, Asn165, Val287, Arg338, and.

**Table 1:** Key active site residues binding to GAG-tetrasaccharide donors in the protein TT16.

Subsite	−2	−1	1	2
HA	R342	R338, Y284, V287	H275, Q274	W166, Q212, N225, R219
CS-A	R342, R338, H275	R219, N264, N225, W166, H214, V287	H275, N165, N225, Y284	V287, W167
CS-C	Y284, N225	R342, E345, R114, W167, V287	R338, N165	N225, Q274, H274, I107, H275, W166
CS-D	R338, R342, E456	(−) Not available	Y284, N165, R338	W166, N225, N224, H275
CS-E	R342, R338	R114, N111, S168, V287, Y284	N165, N225, Q274, H275, Y284	D222, R219, Q212, H214, H275, W510

Trp166 is important for TT16 to recognize and bind to sugar chains of substrates, whereas Tyr284, Asn225, and His275 (which provide the electron transfer basis by containing an imidazole ring in the residue) are located in the same key lyase motif and play essential roles in catalyzing the depolymerization. Furthermore, Ala71 and Arg219 of the lyase TT16 are two site residues located at the inlet and the outlet of the catalytic chamber, respectively, the latter of which can enlarge the catalytic cavity of TT16 in size and can bind to the sulfate groups of CS-E tetrasaccharide substrates to form salt bridges (Figure 7B). Notably, it is different from the Ser71 and Gln219 residues of the *endo*-type enzymes, that is, the HCLaseM and the HCLase_M28 (Sun et al., 2019; Ju et al., 2024), and thus may have led to the rate-limiting step of substrate-entering and product-releasing in an *exo*-type action mode, that is, disaccharide-yielding.

## 5 Conclusion

The GAG lyase TT16 is a protein encoded by the actinomycete *Microbacterium* sp. strain WS15, which is marine-derived and polysaccharide-degrading. The enzyme was classified into the PL8 family, mainly due to sequence similarities with reported enzymes, and it uses multiple GAGs as substrates instead of alginate, dermatan, and heparin. However, TT16 is novel for predominantly yielding unsaturated disaccharides in an *exo*-lytic instead of an *endo*-lytic mode, with the smallest substrate of associated GAG tetrasaccharides. Through further bioinformatic analyses, we discovered that TT16 possesses many properties that enable broad degradation, yet it exhibited limited resistance to sulfated GAGs in a disaccharide-yielding and *exo*-lytic manner. This is due to its unique protein structure (the catalytic cavity), specific key active site residues, and tolerant binding capabilities. This study will provide a tool-like enzyme for preparing GAG disaccharides and will benefit associated enzyme resource identification, rational design, and enzyme improvements.

## Data Availability

The datasets presented in this study can be found in online repositories. The names of the repository/repositories and accession number(s) can be found at: https://www.ncbi.nlm.nih.gov/genbank/, PQ312688 and https://www.ncbi.nlm.nih.gov/genbank/, PQ316084.

## Footnotes

^1^ www.cazy.org

^2^ http://www.ncbi.nlm.nih.gov

^3^ http://www.cbs.dtu.dk/services/

^4^ http://swissmodel.expasy.org/

^5^ https://smart.embl.de

^6^ https://plip-tool.biotec.tu-dresden.de/plip-web/plip/index

^7^ https://plip-tool.biotec.tu-dresden.de/plip-web/plip/index
