# Supplementary figures and images for: A novel exo-lytic and disaccharide-yielding glycosaminoglycan lyase from a marine-derived polysaccharide-degrading actinobacterium Microbacterium sp. strain WS15
Source: Front Microbiol. 2025 Nov 17;16:1680841. doi: 10.3389/fmicb.2025.1680841 (PMC12666371; doi:10.3389/fmicb.2025.1680841)

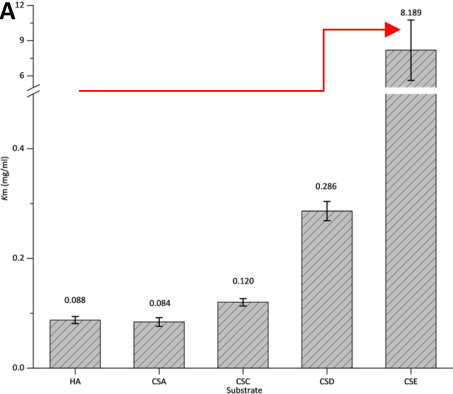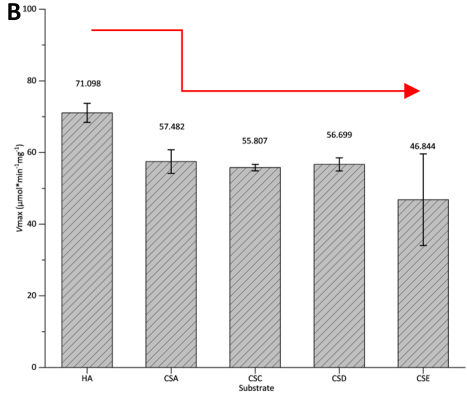

Supplement: Supplementary file 1 [file Image_1.pdf]
